# HER2 expression in breast cancer primary tumours and corresponding metastases. Original data and literature review

**DOI:** 10.1038/sj.bjc.6601881

**Published:** 2004-05-18

**Authors:** J Carlsson, H Nordgren, J Sjöström, K Wester, K Villman, N O Bengtsson, B Ostenstad, H Lundqvist, C Blomqvist

**Affiliations:** 1 Department of Oncology, Radiology and Clinical Immunology, Rudbeck Laboratory, Uppsala University Hospital, SE-751 85 Uppsala, Sweden; 2 Department of Genetics and Pathology, Rudbeck Laboratory, Uppsala University Hospital, Uppsala, Sweden; 3 Department of Oncology, Helsinki University Hospital, Helsinki, Finland; 4 Department of Oncology, University Hospital Umea, Sweden; 5 Department of Oncology, Ullevål University Hospital Oslo, Norway

**Keywords:** breast cancer, c-erb-B2, HER2, metastasis, overexpression, receptors

## Abstract

The aim of this study was to evaluate whether the HER2 expression in breast cancer is retained in metastases. The HER2 expression in primary tumours and the corresponding lymph node metastases were evaluated in parallel samples from 47 patients. The HercepTest was used for immunohistochemical analyses of HER2 overexpression in all cases. CISH/FISH was used for analysis of gene amplification in some cases. HER2 overexpression (HER2-scores 2+ or 3+) was found in 55% of both the primary tumours and of the lymph node metastases. There were only small changes in the HER2-scores; six from 1+ to 0 and one from 3+ to 2+ when the metastases were compared to the corresponding primary tumours. However, there were no cases with drastic changes in HER2 expression between the primary tumours and the corresponding lymph node metastases. The literature was reviewed for similar investigations, and it is concluded that breast cancer lymph node metastases generally overexpress HER2 to the same extent as the corresponding primary tumours. This also seems to be the case when distant metastases are considered. It has been noted that not all patients with HER2 overexpression respond to HER2-targeted Trastuzumab treatment. The stability in HER2 expression is encouraging for efforts to develop complementary forms of therapy, for example, therapy with radionuclide-labelled Trastuzumab.

There is a need for new therapy modalities to improve the survival time for patients with disseminated breast cancer. One approach is to target the antibody Trastuzumab (Herceptin™) to the HER2 receptor when it is overexpressed (Pegram *et al*, 2000; McKeage and Perry, 2002). HER2 (also called ErbB2 or neu) is a transmembrane receptor in the epidermal growth factor (EGF) receptor family. HER2 has no known natural ligand and instead seems to be activated via dimerisation with other receptors in the family: EGFR, HER3 or HER4 (Yarden and Sliwkowski, 2001). HER2 is overexpressed in 25–30% of all breast cancers (Cobleigh *et al*, 1999; Andersson *et al*, 2002) and probably in a higher percentage in the more malignant subgroup that form lymph node or distant metastases (Xu *et al*, 2002; Eccles, 2002; Esteva *et al*, 2002, see also the results in this study).

Binding of Trastuzumab to HER2 blocks growth-stimulating intracellular signalling, decreases the cellular repair capacity after chemo- and radiotherapy, and possibly also improves the apoptosis capacity (Pegram *et al*, 2000; McKeage and Perry, 2002). Thus, the antibody is expected to impart therapeutical effects either alone (Cobleigh *et al*, 1999) or in combination with chemotherapy where taxan seems especially promising (Slamon *et al*, 2001).

However, it has been shown that less than half of the patients with a high expression of HER2 respond to Trastuzumab treatment whether the antibody is given alone or in combination with chemotherapy (Cobleigh *et al*, 1999; Slamon *et al*, 2001; McKeage and Perry, 2002). The reason for resistance to Trastuzumab among tumours that overexpress HER2 is not well clarified, although an increased signalling via the IGF-I receptor has been associated with lack of Trastuzumab response (Lu *et al*, 2001, 2004). Another obvious explanation might be heterogeneity in the expression of HER2 between primary and metastatic tumour cells. It is, for example, feared that the overexpression of HER2 may sometimes be lost in metastases (Thor, 2001). The aim of the present study was to further add to the body of data on this subject and to review some recently published studies.

## MATERIALS AND METHODS

### Patients

Tissue sections were obtained from the Scandinavian Breast Cancer Group study, SBG 9404 (Sjöström *et al*, 2002). All patients with samples from both the primary tumour and from a lymph node metastasis were included. An inclusion criterion for the SBG 9404 study was also the presence of distant metastases. However, tissue samples were not taken from the distant metastases so that these were not available for HER2 analysis. Samples with less good histological quality were excluded if the corresponding FISH analysis was also verified as being of bad quality (see below). High-quality material of both primary tumours and the corresponding metastases were thereafter obtained from 47 patients. All the samples were coded and the analyses were carried out without knowing as to which samples formed pairs of primary tumour and metastasis. The code was then broken and all data were summarised. The patient and tumour characteristics of the 47 analysed patients are shown in Table 1

**Table 1:** Tumour and patient characteristics (*n*=47)

**Characteristics**	**Patients, n (%)**
*Histology*	
Ductal	44 (94)
Lobular	3 (6)
Medullary	0 (0)
	
*Histological grade*	
Grade I	5 (11)
Grade II	30 (64)
Grade III	12 (25)
	
*T-stage*	
pT1	10 (21)
pT2	25 (53)
pT3	11 (23)
pT4	1 (2)
	
*Oestrogen receptor status*	
Positive	23 (49)
Negative	23 (49)
Unknown	1 (2)
Median age (years)	52.2
	
*Menopausal status*	
Premenopausal	3 (6)
Postmenopausal	44 (94)

.

### HER2-staining

The histological and immunohistochemical staining was carried out as previously described (Sjöström *et al*, 2002). Briefly, the tissues were fixed in 4% buffered formalin, processed and embedded in paraffin. Sections, 4-*μ*m thick, were then cut and dried for 12 h at 37°C. The sections were deparaffinised in xylene and rehydrated through graded concentrations of ethanol to distilled water. Incubating the sections in methanol and hydrogen peroxide for 30 min quenched endogenous peroxidase. Immunohistochemical stainings were performed by using the Elite ABC Kit (Vectastain, Vector Laboratories, Burlingame, CA, USA). Blocking serum was applied for 15 min and followed by incubation with rabbit anti-human c-erbB-2 oncoprotein (Dako, Glostrup, Denmark) diluted 1 : 500. Sections were then incubated with the biotinylated secondary antibody and were visualised using the peroxidase substrate 3-amino-9-ethyl-carbazole (AEC) (Sigma A-5754) as chromogen. Finally, the sections were counterstained with Mayer's haematoxylin and mounted with Aquamount (BDH Ltd, Poole, UK).

### HER2-scores

The HER2 expression was scored using the HercepTest criteria. The HER2-score was based on a 0 to 3+ scale. 0 corresponded to tumour cells that were completely negative, 1+ corresponded to faint perceptible staining of the tumour cell membranes, 2+ corresponded to moderate staining of the entire tumour cell membranes and 3+ indicated strong circumferential staining of the entire tumour cell membranes creating a fishnet pattern. The Canadian and the DAKO HercepTest guidelines (Bilous *et al*, 2003) that require more than 10% of the tumour cells to be stained were applied. Owing to the discussions about relevant cutoff levels (Bilous *et al*, 2003), we also considered other levels within the range 10–40%. Cytoplasmic staining was considered nonspecific and was not included in the scoring. As positive controls, we used in-house positive control tissue sections as well as positive control sections supplied by DAKO. In addition, we applied sections from pellets made from the strongly HER2-positive SK-BR-3 breast cancer cells (Bilous *et al*, 2003) that were stained in the same manner. As negative controls, we used HER2-positive breast cancer tissue but excluded the primary antibody. We also used normal tissues that are expected not to express HER2 such as normal tissues near the primary tumours (normal breast glands and connective tissue seen in the same sections as the tumour cells) as internal negative controls. In the metastases sections, we used lymphocytes and the surrounding capsule of the lymph nodes as negative internal controls.

### FISH and CISH

FISH and CISH analyses of HER2 (erbB2) gene amplification were carried out to validate the immunohistochemistry. This verified that the primary tumours with high HercepTest scores (2+ or 3+) were also gene amplified. For a few cases, methodological comparison was also made between FISH and CISH, and an exact match was found. Samples that showed erbB2 gene amplification with FISH also showed amplification with CISH while samples, which did not show amplification with FISH, did not show amplification with CISH.

For the FISH assay, we applied the PathVysion HER-2 DNA Probe Kit (Vysis Inc., Downers Grove, IL, USA). Briefly, formalin-fixed paraffin-embedded tissue specimens were placed on slides. The DNA was denaturated and allowed to hybridise with two fluorescent probes. The chromosome 17-marker probe gave green fluorescence and the erbB2 marker probe gave intense orange fluorescence. The cell nuclei were stained with DAPI giving blue fluorescence. The three fluorescence signals were observed sequentially in the same field of view using a microscope, Leica DMLB, equipped with appropriate filters. The scoring conditions followed the recommendations given by Vysis Inc. Gene amplification was at hand when the number of orange spots per cell was significantly higher than the number of green dots per cell.

The CISH analysis was carried out according to the description by Rummukainen *et al* (2001). Briefly, before hybridisation, the tissue sections were deparaffinised, washed and dried. They were then incubated in buffer at a high temperature in an autoclave and after cooling and rinsing the sections were treated with pepsin, washed, dehydrated and dried. A digoxigenein-labelled HER2 probe was added and the slides were sealed and denaturated, and hybridisation was allowed to take place. The slides were then opened, washed and the HER2 probe was finally detected using antidigoxigenin, goat anti-mouse HRP and DAB. The slides were incubated with DAB enhancer, counterstained with haematoxylin and then analysed in a light microscope.

### Excluded cases

In six patients, the primary tumours and the lymph node metastases deviated severely with respect to their HercepTest score. In all six cases, the samples with lowered HercepTest scores (five metastases and one primary tumour) also had a distorted histological and morphological appearance. In addition, FISH analyses revealed that the samples with lowered HercepTest scores were of such poor quality that the chromosome 17 marker could not be identified. Further, the DAPI stainings were blurry and did not stain the nuclei as expected. We were unable to obtain more material from these patients and they were therefore excluded from the analysis. In the remaining analysed cases (*n*=47), the quality of both the primary tumour and the metastases samples was high.

## RESULTS

The HER2-scores for the analysed 47 primary tumours and the corresponding 47 lymph node metastases are shown in Table 2

**Table 2:** HER2-scores for the analysed primary tumours and the corresponding lymph node metastases (*n*=47)

	**Lymph node metastases HER2-scores**			
**Primary tumour HER2-scores**	**0**	**1+**	**2+**	**3+**
0	13	0	0	0
1+	6	2	0	0
2+	0	0	3	0
3+	0	0	1	22

. There were no changes between the primary tumours and the corresponding lymph node metastases in the majority of cases. Only seven changes were observed. There were six changes from 1+ to 0 and one change from 3+ to 2+ when the metastases were compared to the primary tumours. Only samples scored as 2+ or 3+ were gene amplified as seen from analyses with both FISH and CISH.

The 10% cutoff level was applied. This means that only samples with more than 10% membrane-stained tumour cells were scored 1+, 2+ or 3+ in Table 2. However, the percentage positive tumour cells in all primary tumours and in all metastases were actually in the range 50–100%. Therefore, all cutoff levels below 50% give the same result as reported in Table 2.

The intensity in staining in the primary tumours was, in most cases, either strong (*n*=23) or there was no staining at all (*n*=13). There were not so many cases with intermediate staining intensity, faint staining (*n*=8) and moderate staining (*n*=3). This pattern was similar in the lymph node metastases, which had strong staining in 22 cases, moderate staining in four cases, faint staining in two cases and no staining in 19 cases.

The major results from the HER2-score analyses are summarised in Table 3

**Table 3:** Major results from the HER2-scores analyses (*n*=47)

**HER2-scores characteristics**	**Fraction**	**Percentage**
Primary tumours with 3+	23/47	49
Primary tumours with 2+ or 3+	26/47	55
Lymph node metastases with 3+	22/47	47
Lymph node metastases with 2+ or 3+	26/47	55
Unchanged HER2-scores in lymph node metastases *vs* the primary tumour	40/47	85
Changed HER2-scores in lymph node metastases *vs* the primary tumour	7/47	15
Patients who had 0 or 1+ in primary tumours and changed to 2+ or 3+ in lymph node metastases	0/47	0
Patients who had 2+ or 3+ in primary tumours and changed to 0 or 1+ in lymph node metastases	0/47	0

**Figure 1:**
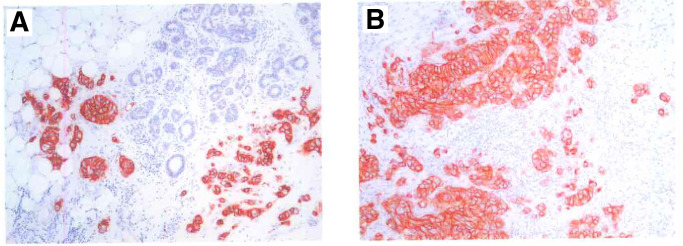
Typical immunohistochemical HER2-stainings. A section from a primary breast tumour (**A**) and a section of a lymph node metastasis (**B**) from the same patient are shown. Both cases were scored 3+.

. It is clear that in the majority of cases, there were no changes between the primary tumours and the corresponding lymph node metastases with respect to HER2 overexpression. Examples of staining patterns for a primary tumour and the corresponding metastasis (which both were scored as 3+) are shown in Figure 1

.

The literature was reviewed for recently published studies on HER2 expression in primary breast tumours and corresponding metastases (see Table 4

**Table 4:** Recent examples from the literature on HER2 expression in primary tumours and the corresponding metastases

**Report**	**Percentage IHC overexpression in primary tumours**	**Percentage IHC overexpression in metastases**	**Comments**
Masood and Bui (2000)	32% (*n*=56)	32% (*n*=56)	2+ or 3+ HercepTest
Shimizu *et al* (2000)	38% (*n*=21)	38% (*n*=21)	+/− scale not HercepTest
Simon *et al* (2001)	24.8% (*n*=125)	21.6% (*n*=125)	2+ or 3+ HercepTest and/or positive FISH
Tanner *et al* (2001)	28% (*n*=46)	28% (*n*=46)	0–3+ scale but not HercepTest
Gancberg *et al* (2002)	29% (*n*=100)	27% (*n*=100)^a^	2+ or 3+ HercepTest
Vincent-Salomon *et al* (2002)	25% (*n*=44)	20.5% (*n*=44)^b^	+/− scale not HercepTest
Tsutsui *et al* (2002)	25% (*n*=76)	25% (*n*=76)	0, +, ++ scale not HercepTest
This article	55% (*n*=47)	55% (*n*=47)	2+ or 3+ HercepTest

a Mainly distant metastases.

b Liver and lung metastases.

Lymph node metastases were analysed in all cases, except in the studies by Vincent-Salomon *et al* (2002) and Gancberg *et al* (2002), where distant metastases were analysed

). Only studies from the last 4 years and in which HercepTest, or a similar immunohistochemical analysis, was applied were included. It is concluded that breast cancer lymph node metastases generally overexpress HER2 in a manner similar to the corresponding primary tumours. In two studies (Vincent-Salomon *et al*, 2002; Gancberg *et al*, 2002), distant metastases were also analysed, and the conclusion was that these also express HER2 as the primary tumours. These conclusions are also supported by previously published FISH studies (e.g. Thor, 2001; Xu *et al*, 2002).

The studies referred to in Table 4 also showed that the similar scorings between the primary tumours and the corresponding metastases were not due to random changes so that a number of HER2 positive primary tumours that converted to HER2 negative metastases were balanced by a similar number of conversions in the other direction. Changes in HER2 expression actually seemed to be extremely rare, although a few cases were reported, and the general conclusion is that the HER2 expression is stable when the metastases are compared to the corresponding primary tumours.

It is noted that the percentage HER2 positive primary tumours and metastases were higher in the present study than in the previously published studies (Table 4). This is difficult to explain since the selections of patient materials were, as far as we know, random in all studies. However, it is possible that the tumours included in our study were, on the average, of more aggressive type than in the other studies. This is not possible for us to judge in detail since we do not have detailed gradings for the different studies (e.g. Elston scores). Our tumours were rather extensive since it is seen from Table 1 that 37 tumours were classified as pT2–pT4 while only 10 were classified as pT1. The aggressive nature of our tumours also stems from the fact that all samples were drawn from patients with subsequent distant metastases. This probably gives a selection for high HER2-expressing tumours. For example, in one study it has been reported that 60% of the studied breast cancer bone marrow metastases were HER2 positive (Braun *et al*, 2001). However, the aim of our study was to compare HER2 expression in primary tumours in relation to the corresponding metastases and for that the considered data are hopefully good enough in all the studies.

## DISCUSSION

The results in the present study and in seven recently published similar studies clearly showed that breast cancer metastases generally overexpress HER2 in a manner similar to the corresponding primary tumours. This stability in HER2 expression seems surprising in the light of the genomic instability that characterises most malignant tumours, including breast cancers. Tumours are formed via multistep carcinogenesis (Bertram, 2000; Onyango, 2002) involving defect onco-, supressor-, cell cycle- and apoptosis-regulating genes. HER2 overexpression can be regarded as overexpression of an oncogene product and erbB2 gene amplification as an oncogene amplification. It is likely that HER2 overexpression is, at least for some tumours, one of the steps in the multistep process towards malignancy and that loss or a decrease in HER2 expression therefore might decrease their growth potential. Tumour cells that lose or downregulate HER2 will then be outgrown in an expanding tumour cell population. They can possibly also be directed towards apoptosis since it has been indicated that changes in HER2 expression can, at least in combination with therapy, modify the route to apoptosis (O'Rourke *et al*, 1998; Yarden and Sliwkowski, 2001).

The arguments given above about the lack of influence of genomic instability on HER2 expression are also of interest when HER2 targeted therapy is considered. It could be expected that an efficient therapy based on targeting of HER2 would tend to induce a selection for cells with low or no HER2 expression. However, as discussed above, such cells might have a decreased growth potential and, during therapy, even be triggered to apoptosis. Thus, it is possible that HER2 is a suitable target also if treatment induced selection is considered.

The observed stability in HER2 expression is also supported by older studies on breast cancer (e.g. Niehans *et al*, 1993) and also by studies on other tumours such as ovarian cancer (Tewari *et al*, 2000), cutaneous squamous carcinomas (Ahmed *et al*, 1997) and urinary bladder carcinomas (Wester *et al*, 2002). However, besides breast cancer no other tumour type has, with respect to this aspect, been extensively analysed by so many research groups, and the conclusions for the other tumour types must so far be considered preliminary.

As mentioned in the Introduction, the treatment of disseminated breast cancer needs improvement and it is necessary to consider new therapy methods. Less than half of the patients with high HER2 expression respond to Trastuzumab treatment (Cobleigh *et al*, 1999; Slamon *et al*, 2001; McKeage and Perry, 2002). The reason for failure is not known, but it has been shown that high insulin-like growth factor-I receptor (IGF-I-R) signalling can counteract the effects of Trastuzumab (Lu *et al*, 2001, 2004). Another explanation might be heterogeneity of expression of HER2 between primary and metastatic tumour cells. The latter is, however, an unlikely explanation considering the results summarised in Table 4.

Targeting against HER2 not only with Trastuzumab but possibly also with radiolabelled Trastuzumab could be an improvement. Radionuclide therapy is a promising modality for treatment of tumours of haematopoietic origin, while the success for treatment of solid tumours has so far been limited (Carlsson *et al*, 2003). However, the promising therapeutic results for haematological tumours give hope that radionuclide therapy will have a breakthrough also for treatment of disseminated cells from solid tumours. Furthermore, resistance induction has so far not been associated with radiation treatment. Antibody or anti-body fragment-mediated targeting of toxins might be another possibility.

New knowledge is continuously emerging with respect to receptor targeting since pharmacokinetics and cellular processing of different types of targeting agent are increasing and the research dealing with molecular design of targeting agents is rapidly expanding. The development of peptides and small proteins with specificity against tumour cells is one strategy (Heppeler *et al*, 2000; Kwekkeboom *et al*, 2000; Park *et al*, 2000). The area of antibody engineering is also rapidly developing and various forms of antibody fragments are being developed such as minimal recognising units, single-chain fragments (scFv) and dimeric scFv (Adams *et al*, 2001; Goldenberg, 2003). Liposomes containing toxic substances and conjugated with targeting agents (Park, 2002; Bohl Kullberg *et al*, 2002) might be of special interest for killing of disseminated tumour cells in the systemic circulation. Thus, there are several possibilities for new and complementary strategies when targeting of disseminated HER2 expressing breast cancer cells are considered.
